# Successful outcome after laparoscopic surgery for sporadic colonic desmoid tumor with β-catenin mutation: a case report

**DOI:** 10.1186/1752-1947-7-100

**Published:** 2013-04-10

**Authors:** Shutaro Gunji, Kenji Kawada, Mayumi Kawada, Suguru Hasegawa, Yoshiharu Sakai

**Affiliations:** 1 Department of Surgery, Kyoto University Graduate School of Medicine, 54 Shogoin- Kawara-cho, Sakyo-ku, Kyoto, 606-8507, Japan; 2 Department of Gastroenterology and Hepatology, Kyoto University Graduate School of Medicine, Kyoto, Japan

**Keywords:** β-catenin mutation, Laparoscopic surgery, Sporadic desmoid tumor

## Abstract

**Introduction:**

Desmoid tumors (also called aggressive fibromatosis) are histologically benign, but have a strong tendency to recur locally after resection. They are rare neoplastic tumors that may occur sporadically or in association with familial adenomatous polyposis caused by a germline mutation in the *adenomatous polyposis coli* gene. The etiology of desmoid tumors is unknown, but their association with a history of abdominal surgery, trauma, and estrogen therapy is well known.

**Case presentation:**

A 36-year-old Asian woman was referred complaining of an abdominal tumor. She had no history of familial adenomatous polyposis, abdominal surgery, trauma or pregnancy. A laparoscopy-assisted right hemicolectomy with a minilaparotomy was conducted for resection of her right-side colon and the anterior wall of her duodenum. The histopathological diagnosis was a desmoid tumor that grew from the transverse mesocolon. Mutational analysis indicated a mutation of the β-catenin gene (*CTNNB1*), consisting of a substitution of threonine for alanine at codon 41. The patient has been followed postoperatively for more than 3 years without any sign of recurrence.

**Conclusion:**

We report a case of sporadic colonic desmoid tumor which was resected by laparoscopic surgery. A successful outcome was achieved because there has been no local recurrence for more than 3 years. The tumor grew from the transverse mesocolon, and harbored a mutation of the *CTNNB1* gene. Mutational analysis of *CTNNB1* gene may play an important role as a prognostic marker of desmoid tumors.

## Introduction

Desmoid tumors (also called aggressive fibromatosis) are uncommon mesenchymal neoplasms with a fibrotic band-like consistency, and are locally aggressive tumors without the potential for distant metastases [1]. They are histologically benign, but have a strong tendency to invade locally and to recur after resection. The incidence of desmoid tumors in the general population is two to four patients per million per year, with a slight female preponderance and a peak incidence in the third and fourth decades [2]. The association of desmoid tumors with familial adenomatous polyposis (FAP) is well established [3]. Trauma, prior surgery, pregnancy and estrogen therapy are other risk factors [4]. Desmoid tumors can develop at any site of the body, but most frequently occur in the extremities of the trunk, abdominal wall, and within the abdomen. Intra-abdominal desmoids predominate in patients with FAP, especially after abdominal surgery and trauma [4].

## Case presentation

**Figure 1:**
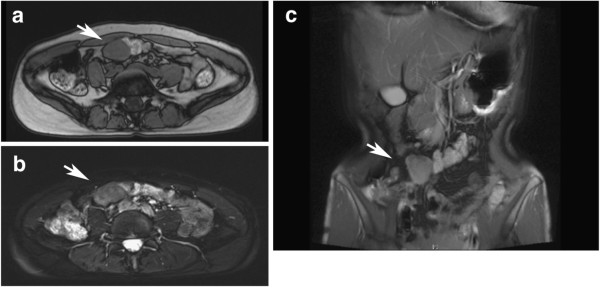
**Magnetic resonance imaging findings showed the desmoid tumor (arrows) with lower signal intensity on T1-weighted image (a) and high signal intensity on T2-weighted image (b and c).** Note that the desmoid tumor was next to contrast-filled transverse colon. (**a** and **b**) Axial plane. (**c**) Coronal plane.

**Figure 2:**
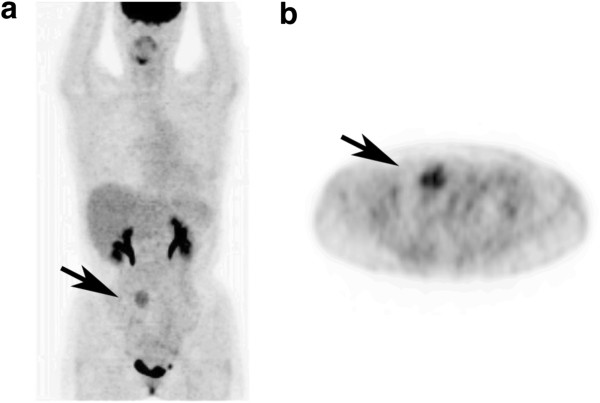
**On the positron emission tomography scan, the heterogeneous uptake (arrows) of **^**18**^**F-fluorodeoxyglucose was seen in the middle-lower abdomen, and no definite metastatic lesion was observed.** (**a**) Coronal plane. (**b**) Axial plane.

A 36-year-old Asian woman was admitted to our hospital with an abdominal mass in the right lower abdomen. The mass was incidentally found by an ultrasonography. On physical examination, the mass was firm, painless, about 3cm in diameter, and not fixed to the abdominal wall. She had no history of abdominal surgery, abdominal injury, pregnancy or FAP. Results of laboratory blood tests, including tumor markers, were within normal ranges. Computed tomography (CT) showed a nodular enhancing soft-tissue mass that was very close to the transverse colon. Magnetic resonance imaging (MRI) showed a tumor with lower signal intensity on T1-weighted imaging and with high signal intensity on T2-weighted imaging (Figure 1). A positron emission tomography CT scan demonstrated heterogeneous uptake of ^18^F-fluorodeoxyglucose into the tumor, and no definite metastatic lesions (Figure 2).

**Figure 3:**
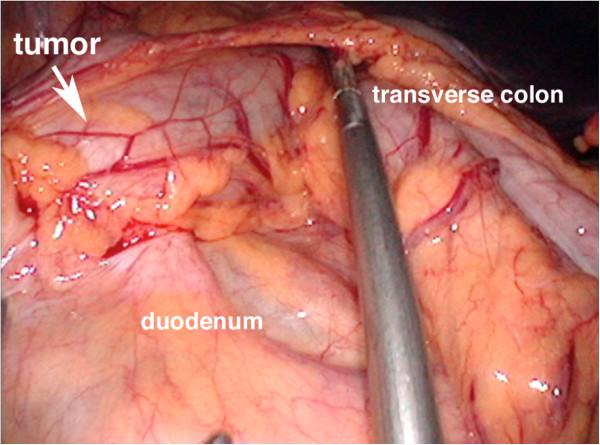
**Intraoperative findings.** Note that the desmoid tumor seemed to originate from the transverse mesocolon, and it involved the duodenum and transverse colon.

Laparoscopic surgery was attempted under a presumptive diagnosis of gastrointestinal stromal tumor (GIST) or other soft-tissue tumor of the transverse colon. The tumor was found to originate from the transverse mesocolon, and the transverse colon and the anterior wall of the duodenum were involved (Figure 3). Duodenal involvement was limited to a small extent. Therefore, in order to resect the tumor en bloc, laparoscopy-assisted right hemicolectomy was conducted with a minilaparotomy for partial resection of the anterior wall of the patient’s duodenum. The anterior wall of her duodenum was closed by a linear stapler. Finally, we confirmed that the patency of the duodenal lumen was preserved.

**Figure 4:**
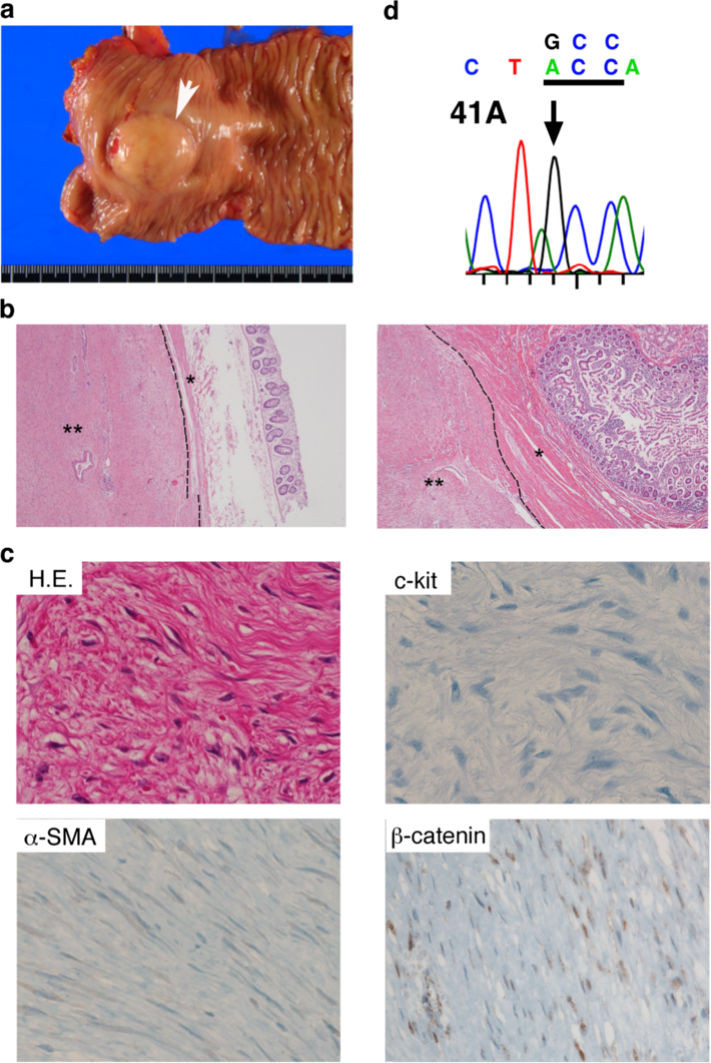
**Resected desmoid tumor.** (**a**) A gross appearance of the tumor (white arrow) seemed like a sub-mucosal tumor. (**b**) Hematoxylin and eosin (H&E, ×40) stain demonstrated that the tumor was directly involved in the muscularis propria of the transverse colon (left), and was very close to the duodenal muscularis propria (right). The broken line indicates the border between the muscularis propria (*) and the tumor (**). (**c**) H&E (×400) stain demonstrated spindle cells proliferation and fibroblast-like cells with bland nuclear morphology (upper left). Immunohistochemical analysis indicated tumor cells were negative for c-Kit (upper right), α-smooth muscle actin (lower left), but positive for β-catenin with nuclear accumulation (lower right). (**d**) Tumor sequence indicated a substitution of GCC (threonine) for ACC (alanine) at codon 41.

The resected specimen yielded a yellowish white solid tumor, measuring 40 × 40 × 25mm (Figure 4a). No necrosis was seen. Microscopically, the tumor directly involved the muscularis propria of the transverse colon, and was very close to the duodenal muscularis propria (Figure 4b). The surgical margin was confirmed to be negative for tumor cells. Pathological examination showed that the tumor was composed of a loose growth of spindle-shaped cells with abundant collagen fibers; cellular atypia and neoplastic change were not observed. Immunohistochemical analysis showed that the tumor cells were negative for c-Kit, CD34, desmin, α-smooth muscle actin, and S-100, but positive for β-catenin with nuclear accumulation (Figure 4c). Based on these findings, the diagnosis of colonic desmoid tumor was established. It has been reported that dysregulation of the Wnt-adenomatous polyposis coli (APC)-β-catenin signaling cascade plays a key role in the pathogenesis of sporadic desmoid tumor [3,4]. Therefore, mutational analyses of the β-catenin gene (*CTNNB1*) and *APC* gene were performed, and then a point mutation of the *CTNNB1* gene was found at codon 41 (a substitution of threonine for alanine; Figure 4d). The patient started oral intake 3 days after the operation, and then was discharged without any complications 2 weeks after the operation. No postoperative adjuvant treatment was done, because she did not want to take it due to her desire to preserve her fertility. Follow-up CT scans have been taken every 6 months after the operation. She has been followed postoperatively for more than 3 years without any signs of recurrence.

## Discussion

Desmoid tumors are rare neoplastic tumors that may occur sporadically or in association with FAP caused by a germline mutation in the *APC* gene. The etiology of desmoid tumors is unknown, but their association with a history of abdominal surgery, trauma, and estrogen therapy (for example oral contraceptive) is well known [4]. Patients with FAP and a family history of desmoid tumors have a 25% chance of developing desmoid tumors. With the increasing use of prophylactic colectomy, desmoid tumors have become an important cause of death in up to 11% of patients with FAP [5]. Intra-abdominal desmoid tumors account for less than 10% of sporadic desmoid tumors, and are particularly common in patients with FAP. The most common groups associated with sporadic desmoid tumors are young women during or after pregnancy. The fibroblast has been shown to exhibit a proliferative response to estrogen. Women with desmoid tumors experience regression of their lesions after attaining menopause [4]. However, in this case, the patient is nulliparous and has none of the risk factors mentioned above.

In this case, the tumor was thought to be a GIST before the operation. The imaging of a desmoid tumor relies mainly on CT and MRI in practice [3]. Desmoid tumors have a heterogeneous signal and inhomogeneous enhancement because of the variable distribution of spindle cells, collagen and myxoid matrix. CT shows a soft-tissue mass of variable attenuation and enhancement. On T2-weighted MRI, signal intensity is usually intermediate between skeletal muscles and subcutaneous fat. Over time, they become more hypointense because of increasing collagen deposition and decreasing cellularity. There are no radiographic characteristics that can reliably distinguish desmoid tumors from other soft-tissue tumors including GIST. Although the imaging may be suggestive, histopathological confirmation is usually necessary for the correct diagnosis.

Sporadic desmoid tumors harbor somatic mutations in either the *APC* gene or *CTNNB1*gene, resulting in β-catenin protein stabilization [6]. In particular, mutations in *CTNNB1* gene have been found in around 85% of sporadic desmoid tumors [7]. Three discrete mutations in the *CTNNB1* gene were identified: 41A (substitution of threonine for alanine at codon 41), 45F (substitution of serine for phenylalanine at codon 45), and 45P (substitution of serine for proline at codon 45). The 41A-mutation was observed in the present case. Mutations in *CTNNB1* gene have been described in a variety of epithelial-originating malignancies [8]. It is very unusual that only three specific mutations have been noted in desmoid tumors, whereas most other neoplasms exhibit a variety of *CTNNB1* mutations at multiple codons [9,10]. These findings may indicate that these specific *CTNNB1* mutations are crucial in desmoid development. It has also been reported that 5-year recurrence-free survival is significantly poorer in 45F-mutated desmoid tumors compared with either 41A-mutation or non-mutated tumors [7]. Multiple studies have demonstrated significant nuclear β-catenin expression in desmoid tumors as evaluated by immunohistochemistry. Little is known concerning the utility of β-catenin expression as a prognostic marker for desmoid tumors or a possible association between β-catenin expression levels and the underlying molecular alterations. Gebert *et al*. reported that desmoid tumors exhibiting increased nuclear expression (defined as >20%) had a significantly higher recurrence rate than those lacking nuclear β-catenin expression [11], whereas Lazar *et al*. reported that decreased nuclear β-catenin staining intensity was significantly associated with recurrence [7]. Further investigations are needed in a prospective way to evaluate the potential causal relationship between *CTNNB1* gene mutation, β-catenin expression, and desmoid recurrence.

Treatment choice is dictated by anatomic considerations. If feasible, surgical resection with a wide margin is the mainstay of treatment for symptomatic desmoid tumors. However, recurrence after surgical resection is common (19% to 77%) [4]. Treatment options for desmoid tumors include observation, surgical resection, radiotherapy or systemic therapy. Radiation has been reported to be comparable to surgery and is also useful as an adjuvant treatment to reduce the risk of local recurrence [12]. Systemic therapies include cytotoxic agents (for example, anthracycline), nonsteroidal anti-inflammatory agents (for example, celecoxib and sulindac), anti-estrogen hormonal agents (for example, tamoxifen), and molecular targeted agents (for example, imatinib and sorafenib) [4]. Garbay *et al*. recently reported from a retrospective study of 62 patients with recurrent and/or unresectable desmoid tumors that anthracycline-containing regimens are significantly related to a higher response rate [13]. Heinrich *et al*. reported that imatinib can be an active agent in the treatment of aggressive desmoid tumors, possibly through inhibition of PDGFRB kinase activity [14]. In this case, she did not receive any adjuvant treatment because she desired to preserve her fertility. Fortunately, she has not exhibited any recurrence for more than 3 years. Burke *et al*. reported that the recurrence rate of intra-abdominal desmoid tumors was significantly lower in patients without FAP than in patients with FAP [15]. In addition, mutational analysis revealed that 41A-mutation in the *CTNNB1* gene had occurred in this case, which may result in a better prognosis [7]. Further studies pertaining to clinical practice are required.

## Conclusion

We present a rare case of sporadic colonic desmoid tumor of the transverse mesocolon in a patient without any history of FAP, abdominal surgery, trauma, or pregnancy. A laparoscopy-assisted right hemicolectomy with a minilaparotomy was conducted for the resection of the patient’s right-sided colon and the anterior wall of her duodenum. Mutational analysis indicated the desmoid tumor harbored a mutation of the *CTNNB1* gene, consisting of a substitution of threonine for alanine at codon 41. The patient has not exhibited any evidence of recurrence for more than 3 years. Mutational analysis of the *CTNNB1* gene may play an important role as a prognostic marker of desmoid tumors.

## Consent

Written informed consent was obtained from the patient for publication of this case report and any accompanying images. A copy of the written consent is available for review by the Editor-in-Chief of this journal.

## Competing interests

The authors declare that they have no competing interests.

## Authors’ contributions

KK analyzed and interpreted the patient data. SG and KK were major contributors in writing the manuscript. MK performed the mutational analyses of *CTNNB1* and *APC* genes. SH and YS gave useful comments on this manuscript. All authors read and approved the final manuscript.
